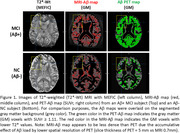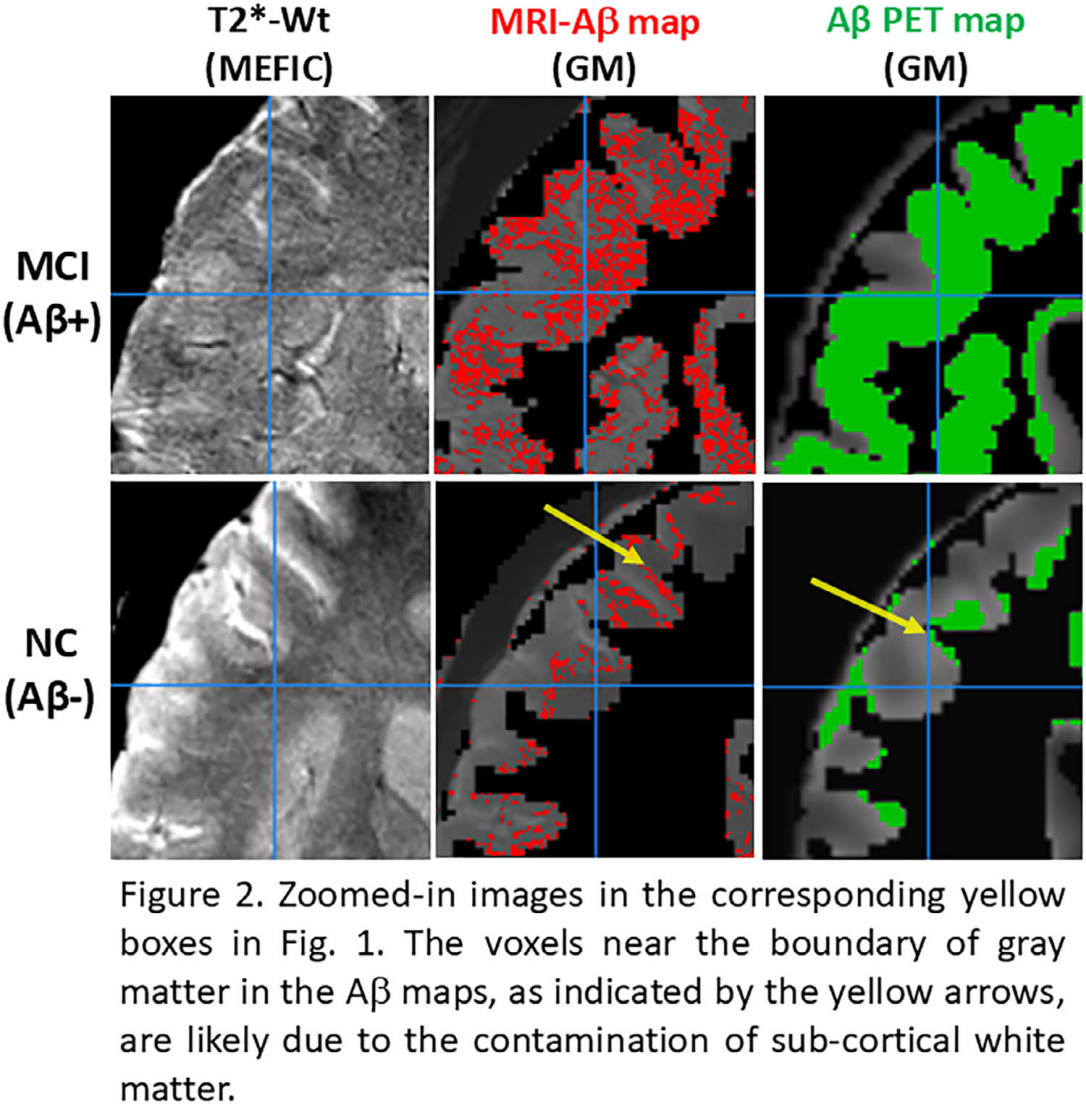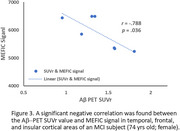# Imaging Aß Plaques in living Human Brains with 3T MRI

**DOI:** 10.1002/alz70856_103011

**Published:** 2025-12-25

**Authors:** Qing Yang, Rommy Elyan, Ran Pang, Sangam Kanekar, Jens Will, Deepak Kalra, Paul Eslinger, Prasanna Karunanayaka, Jianli Wang

**Affiliations:** ^1^ Pennsylvania State University College of Medicine, Hershey, PA, USA

## Abstract

**Background:**

Diagnosis and treatment of AD relies on imaging of beta amyloid (Aβ) plaques in the brain using PET with radiotracer injection, and brain structural changes using MRI—two imaging modalities with extremely high costs. We have demonstrated that Aβ‐plaques can be seen with MRI in high spatial resolution images in brain histological samples using a high field (7T and above), and with long acquisition times (7+ hours), which is too long for clinical human imaging studies. To address this technological challenge, we explored the feasibility of an MRI method (multiple‐echo frequency‐domain image contrast (MEFIC), which could improve signal‐noise‐ratio for high spatial resolution while optimizing T2* contrast, to image Aβ‐plaques in living human brains at 3T.

**Method:**

Eighteen mild cognitive impaired (MCI) and twelve normal (CN) subjects took part in the study. MEFIC images were acquired on a Siemens Prisma‐Fit 3T scanner with a 64‐channel head coil in a resolution of 0.4 x 0.4 x 0.7 mm^3^ and scan time of 12:49. Aβ‐PET scan was conducted on a Siemens Biograph mCT 20 PET/CT scanner with Florbetapir. Clinica software was used to calculate the standardized uptake value ratio (SUVr) with SUVr ≥ 1.11 as positive. PET and MEFIC images were co‐registered in the patients’ native space with SPM12.

**Result:**

Figures 1‐2 show evident correspondence between MRI‐Aβ map (MEFIC) and the PET‐Aβ (SUVr) map from one Aβ+ (MCI) and one Aβ‐ (CN) subjects. The prominent contrast between gray matter (GM) and white matter (WM) in the Aβ‐ subject was diminished in the Aβ+ subject mainly due to the Aβ load in the GM. MEFIC imaging provides a greater (10x) T2* contrast to noise ratio over conventional T2* imaging, which allows for high spatial resolution in‐vivo brain imaging at 3T. There was a significant negative correlation between the Aβ‐PET SUVr value and MEFIC signal in cortical GM (Figure 3).

**Conclusion:**

We have shown that Aβ‐plaque can be detected in T2* MRI images with MEFIC in the human brain at 3T. We have confirmed that MRI T2* in GM negatively correlates to SUVr of Aβ PET.